# Supplementary material for: Barriers and facilitators to integrating tuberculosis treatment into community pharmacies for people with TB/HIV in Kampala, Uganda: A qualitative study
Source: PLOS Glob Public Health. 2025 Nov 21;5(11):e0004951. doi: 10.1371/journal.pgph.0004951 (PMC12637944; doi:10.1371/journal.pgph.0004951)
Supplement: S1 File — (PDF) [file pgph.0004951.s001.pdf]

Zoho Sign Document ID: 304BCE2B-EXZUO4ICLZFCC_BXUHVDFMXDZGFOLRVDADTGVEAZ6UY

Appendix 1. In-depth Interviews (IDI) among people with TB/HIV

**Study title**	Integrating Tuberculosis Treatment into Community Pharmacies to improve TB/HIV outcomes in Uganda
**Short title**	Community Pharmacy Tuberculosis Treatment (COPHAT) study
**Sponsor**	National Institutes of Health (NIH).
**Protocol version and date**	Version #1, April 2024

Dear Sir/madam

Thank you for accepting to participate in this interview. This interview will focus on the integration of TB treatment into community pharmacies for people with TB/HIV. The study proposes to provide TB treatment at community pharmacies along with ART for people with TB and HIV. In this interview, you will express your opinion regarding the barriers and facilitators to integrating TB treatment into community pharmacies. Before we get started, I would like to show and explain to you the proposed strategy as shown in Figure 2 below.

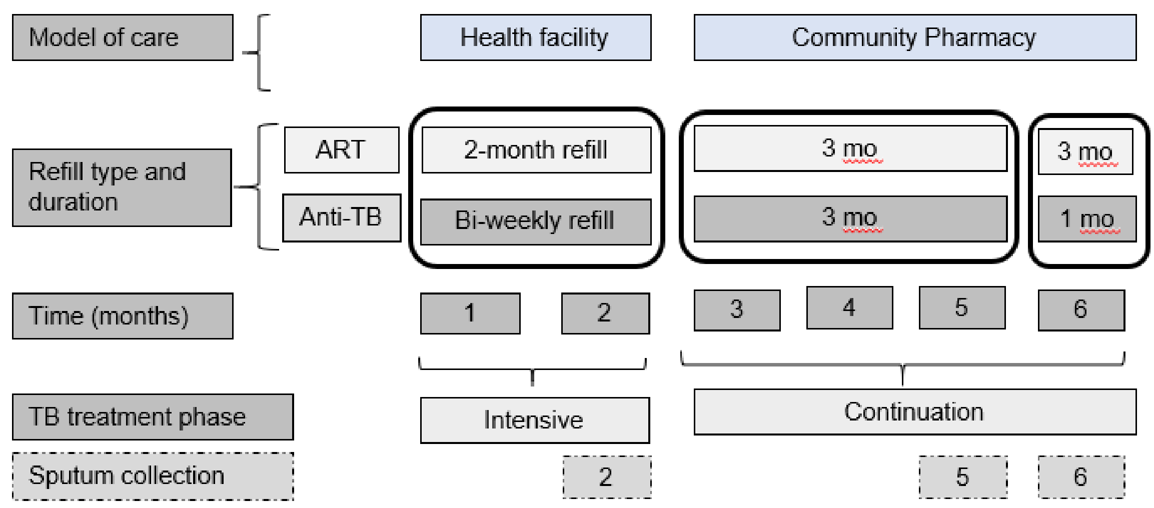

Figure 2. Hypothetical diagram showing the integration of TB treatment into community pharmacies. Explanation: People with TB/HIV will be assessed for stability on all TB clinic visits in the first 2 months; provided with standardized TB counseling and health education, and adherence assessment and support; receive sputum smear follow-up exams at months 2, 5, and 6 if with bacteriological confirmed PTB. ART will be refilled for 2 months while anti-TB medications are refilled bi-weekly or monthly during the first 2 months. At month 2, people with TB/HIV are randomized to either community pharmacy or treatment as usual. In the community pharmacies, ART and anti-TB medications are refilled once for 3 months and once for 1 month.

Duration of interview

1. Start time of KII _____________
2. End time of KII ______________

INFECTIOUS DISEASES INSTITUTE
RESEARCH ETHICS COMMITTEE (IDIREC)
APPROVED
VALID UNTIL
29 Apr 2025

Zoho Sign Document ID: 304BCE2B-EXZUO4ICLZFCC_BXUHVDFMXDZGFOLRVDADTGVEAZ6UY

Participant characteristics

Level of participant	Participant type
Health facility<br>1. Kisenyi<br>2. Kawaala<br>3. Komamboga<br>4. Kiswa<br>5. Kitebi<br>6. Kisugu	Age: _____________ (completed years)<br><br>Sex<br>1. Male<br>2. Female
Level of education<br>1. None<br>2. Primary<br>3. Secondary/tertiary and higher	Employment status<br>1. None<br>2. Self-employed<br>3. Formal employment

Section 1. Intervention characteristics

This study proposes to refill both ART and TB treatments through community pharmacies targeting people with TB/HIV. Share with me the aspects of community pharmacies that might make the proposed strategy a good fit.

Probing questions

- Whether community pharmacy location make it more desirable than routine care?
- Whether the time of medication pick-ups make community pharmacies a better approach than routine care?
- Whether the proposed frequency of ART and TB treatment refills would be considered more important?

Section 2. Individual characteristics

Tell me the kinds of people with TB/HIV who might benefit (or not benefit) from the integration of TB treatment into community pharmacies.

Probing questions.

- Whether stability on ART should be considered a requirement?
- Whether adherence to appointment-keeping would be considered a requirement?
- Whether the strategy should be considered for distant persons or all?
    - If yes, how far or near should the beneficiaries be?
- Are treatment support systems needed?
    - If yes, what type of treatment support systems will be needed?
    - If yes, why, and for whom?
- What aspects of the intervention might require modification and why?

INFECTIOUS DISEASES INSTITUTE
RESEARCH ETHICS COMMITTEE (IDIREC)
APPROVED
VALID UNTIL
29 Apr 2025

Zoho Sign Document ID: 304BCE2B-EXZUO4ICLZFCC_BXUHVDFMXDZGFOLRVDADTGVEAZ6UY

Section 3. Inner setting

Let us discuss some of the factors that might influence the integration of TB treatment into community pharmacies. In your opinion, what factors at the health facility and community pharmacy levels will either positively or negatively affect the integration?

Probe the following:

- Whether community pharmacy health worker attributes such as qualifications, experiences, and training would be important?
- Whether health workers would consider integration as an important intervention
  - If important, why is that so? If not important, what are the reasons?

Section 4. Outer setting

Let us talk about community-level factors that might either positively or negatively affect the integration of TB treatment into community pharmacies. What do you think are some of the benefits of community pharmacy refills when compared to health facility refills?

Probe the following

- Whether community pharmacy refills would effectively deal with self and community stigma, and discrimination?
- Whether the opening hours would be considered important? If so, how? And, what is the preferred opening hour?
- Whether community pharmacy refills will be preferred over health facility refills, and the reasons?

Section 5. Processes

Let us talk about the processes/steps involved in integrating TB treatment into community pharmacies. Tell me how you feel the integration should be done.

Probe the following:

- Timing of the TB treatment integration.
  - Whether at treatment initiation, after 2 weeks, after 1 month, or after 2 months?
  - Reason for the choice of timing?
- Frequency of refills.
  - Monthly, after every 2 months, or after every 3 months?
  - Reasons for choice of timing?
- How sputum smear follow-up exams should be done.
  - Whether through pharmacy-only visits? Whether within the community pharmacies? Whether at the community pharmacy or the TB clinic?
  - How viral load data can be captured?
- Logistical support needs.
  - Whether supervision is necessary? If necessary, by whom and what frequency?
- Whether training on integration is important for health workers (TB focal persons, ART focal persons, and community pharmacy workers)?

INFECTIOUS DISEASES INSTITUTE
RESEARCH ETHICS COMMITTEE (IDIREC)
APPROVED
VALID UNTIL
29 Apr 2025

Zoho Sign Document ID: 304BCE2B-EXZUO4ICLZFCC_BXUHVDFMXDZGFOLRVDADTGVEAZ6UY

Appendix 2. In-depth Interviews (IDI) among health workers (TB focal persons, ART focal persons, and community pharmacy health workers)

**Study title**	Integrating Tuberculosis Treatment into Community Pharmacies to improve TB/HIV outcomes in Uganda
**Short title**	Community Pharmacy Tuberculosis Treatment (COPHAT) study
**Sponsor**	National Institutes of Health (NIH).
**Protocol version and date**	Version #1, April 2024

Dear Sir/madam

Thank you for accepting to participate in this interview. This interview will focus on the integration of TB treatment into community pharmacies for people with TB/HIV. The study proposes to provide TB treatment at community pharmacies along with ART for people with TB and HIV. In this interview, you will express your opinion regarding the barriers and facilitators to integrating TB treatment into community pharmacies. Before we get started, I would like to show and explain to you the proposed strategy as shown in Figure 2 below.

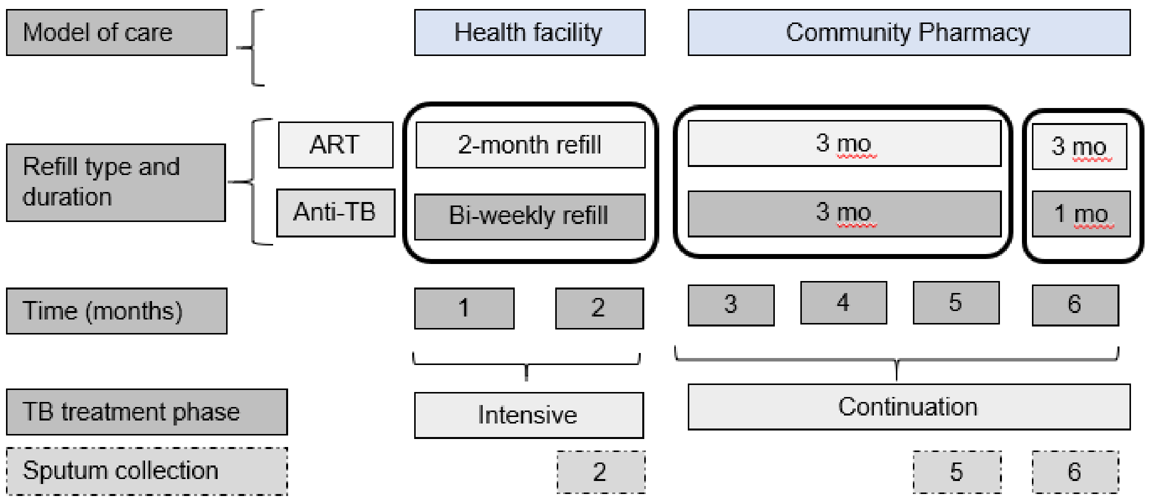

Figure 2. Hypothetical diagram showing the integration of TB treatment into community pharmacies. Explanation: People with TB/HIV will be assessed for stability on all TB clinic visits in the first 2 months; provided with standardized TB counseling and health education, and adherence assessment and support; receive sputum smear follow-up exams at months 2, 5, and 6 if with bacteriological confirmed PTB. ART will be refilled for 2 months while anti-TB medications are refilled bi-weekly or monthly during the first 2 months. At month 2, people with TB/HIV are randomized to either community pharmacy or treatment as usual. In the community pharmacies, ART and anti-TB medications are refilled once for 3 months and once for 1 month.

Duration of interview

1. Start time of KII ______________
2. End time of KII _______________

INFECTIOUS DISEASES INSTITUTE
RESEARCH ETHICS COMMITTEE (IDIREC)
APPROVED
VALID UNTIL
29 Apr 2025

Zoho Sign Document ID: 304BCE2B-EXZUO4ICLZFCC_BXUHVDFMXDZGFOLRVDADTGVEAZ6UY

Participant characteristics

Level of participant	Participant type
Health facility<br>1. Kisenyi<br>2. Kawaala<br>3. Komamboga<br>4. Kiswa<br>5. Kitebi<br>6. Kisugu	Age: ____________ (completed years)<br><br>Sex<br>1. Male<br>2. Female
Level of education<br>1. None<br>2. Primary<br>3. Secondary/ tertiary and higher	Employment status<br>1. None<br>2. Self-employed<br>3. Formal employment

Section 1. Intervention characteristics

This study proposes to refill both ART and TB treatments through community pharmacies targeting people with TB/HIV. Share with me the aspects of community pharmacies that might make the proposed strategy a good fit.

Probing questions

- Whether the location of a community pharmacy make it more desirable than routine care?
- Whether the time of medication pick-ups make community pharmacies a better approach than routine care?
- Whether the proposed frequency of ART and TB treatment refills would be considered more important?

Section 2. Individual characteristics

Tell me the kinds of people with TB/HIV who might benefit (or not benefit) from the integration of TB treatment into community pharmacies.

Probing questions.

- Whether stability on ART should be considered a requirement?
- Whether adherence to appointment-keeping would be considered a requirement?
- Whether the strategy should be considered for distant persons or all?
    - If yes, how far or near should the beneficiaries be?
- Whether treatment support system is needed?
    - If yes, what type of treatment support systems will be needed?
    - If yes, why, and for whom?
- What aspects of the intervention might require modification and why?

INFECTIOUS DISEASES INSTITUTE
RESEARCH ETHICS COMMITTEE (IDIREC)
APPROVED
VALID UNTIL
29 Apr 2025

Zoho Sign Document ID: 304BCE2B-EXZUO4ICLZFCC_BXUHVDFMXDZGFOLRVDADTGVEAZ6UY

Section 3. Inner setting

Let us discuss some of the factors that might influence the integration of TB treatment into community pharmacies. In your opinion, what factors at the health facility and community pharmacy levels will either positively or negatively affect the integration?

Probe the following:

- Whether community pharmacy health worker attributes such as qualifications, experiences, and training would be important?
- Whether health workers would consider integration as an important intervention
  - If important, why is that so?
  - If not important, what are the reasons?

Section 4. Outer setting

Let us talk about community-level factors that might either positively or negatively affect the integration of TB treatment into community pharmacies. What do you think are some of the benefits of community pharmacy refills when compared to health facility refills?

Probe the following

- Whether community pharmacy refills would effectively deal with self and community stigma, and discrimination?
- Whether the opening hours would be considered important? If so, how? And, what is the preferred opening hour?
- Whether community pharmacy refills will be preferred over health facility refills, and the reasons?

Section 5. Processes

Let us talk about the processes/steps involved in integrating TB treatment into community pharmacies. Tell me how you feel the integration should be done.

Probe the following:

- Initial planning, organizing, coordination, and evaluation needed
- Timing of TB treatment integration
  - Whether at treatment initiation, after 2 weeks, after 1 month, or after 2 months?
  - Reason for the choice of timing?
- Frequency of refills
  - Monthly, after every 2 months, or after every 3 months?
  - Reasons for choice of timing?
- Recording of data into existing health management information systems
- How sputum smear follow-up exams should be done.
  - Whether through pharmacy-only visits?
  - Whether within the community pharmacies?

INFECTIOUS DISEASES INSTITUTE
RESEARCH ETHICS COMMITTEE (IDIREC)
APPROVED
VALID UNTIL
29 Apr 2025

Zoho Sign Document ID: 304BCE2B-EXZUO4ICLZFCC_BXUHVDFMXDZGFOLRVDADTGVEAZ6UY

- How the recording of treatment outcome data should be handled
    - Whether at the community pharmacy or the TB clinic?
    - How viral load data can be captured?
- Logistical support needs
    - Whether supervision is necessary? If necessary, by whom and what frequency?
- Whether training on integration is important for health workers (TB focal persons, ART focal persons, and community pharmacy workers)?

INFECTIOUS DISEASES INSTITUTE
RESEARCH ETHICS COMMITTEE (IDIREC)
APPROVED
VALID UNTIL
29 Apr 2025
