# Supplementary material for: Barriers and facilitators to integrating tuberculosis treatment into community pharmacies for people with TB/HIV in Kampala, Uganda: A qualitative study
Source: PLOS Glob Public Health. 2025 Nov 21;5(11):e0004951. doi: 10.1371/journal.pgph.0004951 (PMC12637944; doi:10.1371/journal.pgph.0004951)
Supplement: S2 File — (PDF) [file pgph.0004951.s002.pdf]

Additional File 2: Consolidated criteria for reporting qualitative studies (COREQ) guide

Developed from:

Tong A, Sainsbury P, Craig J. Consolidated criteria for reporting qualitative research (COREQ): a 32-item checklist for interviews and focus groups. International Journal for Quality in Health Care. 2007. Volume 19, Number 6: pp. 349 – 357

Item No	Guide Questions/Description	Reported on Page #
**Domain 1: Research team and reflexivity**		
**Personal Characteristics**		
1. Interviewer/ facilitator	Which author/s conducted the interview or focus group?	Pg 5-6
2. Credentials	What were the researcher's credentials? E.g., PhD, MD	Pg 5-6
3. Occupation	What was their occupation at the time of the study?	Pg 5-6
4. Gender	Was the researcher male or female?	Pg 5-6
5. Experience and training	What experience or training did the researcher have?	Pg 5-6
**Relationship with participants**		
6. Relationship established	Was a relationship established prior to study commencement?	Pg 7
7. Participant knowledge of the interviewer	What did the participants know about the researcher? e.g. personal goals, reasons for doing the research?	Pg 7
8. Interviewer characteristics	What characteristics were reported about the interviewer/facilitator? e.g. Bias, assumptions, reasons and interests in the research topic	Pg. 5-6
**Domain 2: study design**		
**Theoretical framework**		
9. Methodological orientation and Theory	What methodological orientation was stated to underpin the study? e.g. grounded theory, discourse analysis, ethnography, phenomenology, content analysis	Pg 5
**Participant selection**		
10. Sampling	How were participants selected? e.g., purposive, convenience, consecutive, snowball	Pg 5

Item No	Guide Questions/Description	Reported on Page #
11. Method of approach	How were participants approached? e.g., face-to-face, telephone, mail, email	Pg 6
12. Sample size	How many participants were in the study?	Pg 7
13. Non-participation Setting	How many people refused to participate or dropped out? Reasons?	N/A
14. Setting of data collection	Where was the data collected? e.g., home, clinic, workplace	Pg 6
15. Presence of nonparticipants	Was anyone else present besides the participants and researchers?	N/A
16. Description of sample	What are the important characteristics of the sample? e.g. demographic data, date	Pg 5-6
**Data collection**		
17. Interview guide	Were questions, prompts, and guides provided by the authors? Was it pilot tested?	Pg 7-8
18. Repeat interviews	Were repeat interviews carried out? If yes, how many?	N/A
19. Audio/visual recording	Did the research use audio or visual recording to collect the data?	Pg 6
20. Field notes	Were field notes made during and/or after the interview or focus group?	Pg 6
21. Duration	What was the duration of the interviews or focus group?	Pg 6
22. Data saturation	Was data saturation discussed?	Pg 7
23. Transcripts returned	Were transcripts returned to participants for comment and/or correction?	N/A
**Domain 3: analysis and findings**		
**Data analysis**		
24. Number of data coders	How many data coders coded the data?	Pg 7
25. Description of the coding tree	Did the authors provide a description of the coding tree?	N/A
26. Derivation of themes	Were themes identified in advance or derived from the data?	Pg 6-7
27. Software	What software, if applicable, was used to manage the data?	7

Item No	Guide Questions/Description	Reported on Page #
28. Participant checking	Did participants provide feedback on the findings?	Pg 7-8
**Reporting**		
29. Quotations presented	Were participant quotations presented to illustrate the themes/findings? Was each quotation identified? e.g., participant number	Pg 11-20
30. Data and findings consistent	Was there consistency between the data presented and the findings?	Pg 11-20
31. Clarity of major themes	Were major themes clearly presented in the findings?	Pg 20
32. Clarity of minor themes	Is there a description of diverse cases or a discussion of minor themes?	Pg 20
