# Supplementary material for: Barriers and facilitators to integrating tuberculosis treatment into community pharmacies for people with TB/HIV in Kampala, Uganda: A qualitative study
Source: PLOS Glob Public Health. 2025 Nov 21;5(11):e0004951. doi: 10.1371/journal.pgph.0004951 (PMC12637944; doi:10.1371/journal.pgph.0004951)
Supplement: Attachment — Submitted filename: Responses to Reviewers.docx [file pgph.0004951.s005.docx]

**Responses to review comments**

**Reviewer #1:**

1. The manuscript is technically sound, the author followed the guidelines in submitting their manuscript. The methods used to select the participants is clearly mentioned although some were purposively selected which introduces selection bias but the author has mentioned this as a limitation in the study.

**Response. Thank you for the encouraging feedback and for taking the time to review our manuscript. We agree that some of the participants have been purposively selected and this potentially introduces selection bias but this was acknowledged as a limitation.**

2. The information reads well but the level of education or cadres for the community pharmacy health care practitioners is not clear. This would further inform the reader about the competences and knowledge the community pharmacy health care practitioners have about TB.

**Response: All community pharmacy HCPs were trained in nursing or pharmacy and held a bachelor’s degree in their respective fields. To clarify the distribution of participant characteristics, we have presented a revised Table 2 on page 9-10 as a cross-tab by participant type. The revised table highlights the distribution of each variable across groups. Our revised description of the findings in Table 2 reads as follows:**

**“Table 2 presents the characteristics of the participants included in the study displayed by the type of participants. We enrolled 47 individuals: six TB focal persons, six HIV focal persons, nine community pharmacy HCPs, three MoH experts, and 23 people with TB/HIV. The mean age varied by professional cadre, from 30.7 (standard deviation [SD]=7.0) years among community pharmacy HCPs to 47.7 (SD=3.8) years among MoH experts. Most of the HCPs were younger than 35 years, while people with TB/HIV were more often 35 years or older. Sex distribution was nearly balanced across the groups, though people with TB/HIV were slightly more often male. Educational attainment and employment differed, with all HCPs formally employed and highly educated, while people with TB/HIV reported lower levels of education and were mainly self-employed or unemployed.”**

**Reviewer #2:**

The manuscript presents a theory-informed qualitative study designed to explore the feasibility of integrating tuberculosis treatment into community pharmacies for TB/HIV patients in Kampala, Uganda. Through interviews with patients, healthcare providers from six public health facilities, and Ministry of Health experts, the study identified facilitators and barriers to integration. The findings suggest that integrating TB treatment into community pharmacies offers advantages such as convenience, privacy, and cost-effectiveness, but also faces challenges like inadequate pharmacy infrastructure, insufficient staff training, and unclear operational procedures. I have some comments for future improvement.

1. In the “Methods” section, the authors mention using the saturation principle to determine the sample size, but the description of how to specifically operate this principle is not detailed. For example, the text mentions “Specifically, this was observed when ≤5% of the information was new, conditional on a base size of 20 and a run length of 15,” but it does not explain how to judge the novelty of the information and how to decide whether saturation has been reached based on this standard in actual operation.

**Response: Thank you for this comment. To clarify, the saturation principle was operationalized by assessing the proportion of new information emerging from each additional interview. After the initial base size of 20 interviews, we tracked the occurrence of novel concepts or themes in subsequent interviews. Saturation was considered achieved when ≤5% of the information obtained from new interviews was novel, i.e., when most ideas were already captured in previous interviews. This process allowed us to systematically judge the novelty of information and determine when data collection could stop. We have revised the Methods section to reflect this more detailed description. The revised text on page 7 reads as follows:**

**“We determined the sample size using the saturation principle, defined as the point at which additional interviews yield minimal new information. Specifically, after conducting an initial base of 20 interviews, each subsequent interview was assessed for new concepts or themes. Saturation was considered achieved when ≤5% of the information from new interviews was novel, based on a run length of 15, indicating that most themes had already been captured in previous interviews. This approach allowed us to systematically evaluate when sufficient data had been collected.”**

2. In the “Methods” section, the authors mention using framework analysis for data analysis, but the description of the coding process is not clear. For example, the text mentions “JI and RK independently reviewed 5-7 transcripts to familiarize themselves with the data, coded the transcripts by flagging important texts, identified emerging patterns, and jointly developed a preliminary codebook by consensus,” but it does not provide a detailed explanation of how to determine which texts are “important” and how to identify “emerging patterns.”

**Response: Thank you for this comment. The coding and analysis were guided by the CFIR framework, which also informed the identification of important texts within the transcripts. We have revised the text on page 7 to provide more detail, and it now reads:**

**"JI and RK independently reviewed 5–7 transcripts to familiarize themselves with the data, followed by independent coding of the transcripts by flagging texts considered important based on CFIR domains. Emerging patterns were identified from these texts, guided by CFIR constructs, and a preliminary codebook was jointly developed by consensus."**

3. In the “Discussion” section, the authors discuss the research results, but the interpretation of some key results is not deep enough. For example, the text mentions “Enhanced anonymity provided by community pharmacies emerged as a key facilitator of TB treatment integration,” but it does not further explore how this anonymity specifically affects patients' behavior and psychology and the potential mechanisms of its impact on treatment outcomes.

**Response: Thank you for this insightful comment. We agree that the role of anonymity requires deeper interpretation. We have now expanded the discussion to explain how anonymity in community pharmacies influences patients’ behavior, psychology, and ultimately treatment outcomes. Specifically, we describe how anonymity reduces anticipated and enacted stigma, minimizes fear of disclosure, and promotes care-seeking and retention in treatment. The new text on page 21 reads as follows:**

**“Enhanced anonymity in community pharmacies reduces the fear of stigma and unwanted disclosure, making people with TB/HIV more comfortable to seek care and continue treatment. This sense of privacy fosters psychological safety, encourages timely treatment initiation, and normalizes TB/HIV care alongside other healthcare needs. Together, these mechanisms help strengthen adherence and continuity of care, ultimately improving treatment outcomes.”**

4. In the “Conclusion and recommendation” section, the authors propose research recommendations, but the recommendations are not specific enough. For example, the text mentions “context-specific interventions that are developed in collaboration with key stakeholders, address barriers such as eligibility criteria refinement, enhanced pharmacy HCP training, and financial incentives, and leverage facilitators like policy support and stakeholder readiness, are needed,” but it does not provide detailed suggestions on how to implement these interventions. It is suggested that the authors supplement more specific implementation suggestions.

**Response: Thank you for this comment. We acknowledge the need for more specific guidance on implementing the recommended interventions. Our study is intended to inform the adaptation of implementation strategies using a Human-Centered Design (HCD) methodology, which will allow for context-specific refinement in collaboration with key stakeholders described in the present study. These strategies will subsequently be tested in a pilot Type 2 hybrid effectiveness-implementation trial to assess both their feasibility, fidelity, and preliminary effectiveness. We have revised the text in the 'Conclusion and Recommendations Section' to reflect these next steps, highlighting that the proposed interventions will be operationalized through stakeholder-driven design and systematically evaluated in a subsequent implementation trial. Our revised text on page 24 of the conclusion and recommendations section reads as follows:**

**"Context-specific interventions addressing barriers such as eligibility criteria refinement, enhanced pharmacy healthcare provider training, and financial incentives, and leveraging facilitators like policy support and stakeholder readiness, will be developed and refined using a Human-Centered Design approach in collaboration with key stakeholders. These strategies will subsequently be piloted in a Type 2 hybrid effectiveness-implementation trial to evaluate their feasibility, fidelity, acceptability, and preliminary effectiveness."**
